# Identification of CDC42 Effectors Operating in FGD1-Dependent Trafficking at the Golgi

**DOI:** 10.3389/fcell.2019.00007

**Published:** 2019-02-04

**Authors:** Mikhail Egorov, Roman Polishchuk

**Affiliations:** Telethon Institute of Genetics and Medicine, Naples, Italy

**Keywords:** Aarskog-Skott syndrome, FGD1, post-Golgi transport, signaling, GEF

## Abstract

Loss of function mutations in the *FGD1* gene cause a rare X-linked disease, faciogenital dysplasia (FGDY, also known as Aarskog-Skott syndrome), which is associated with bone and urogenital abnormalities. The *FGD1* gene encodes à CDC42-specific guanine nucleotide exchange factor. The mutations are frequently located in the DH module of FGD1 preventing its transformation to the active form. We previously reported that Golgi-associated FGD1 regulates post-Golgi transport of some conventional and bone-specific proteins in a CDC42-dependent manner. However, the downstream targets of FGD1/CDC42 signaling that operate to support transport from the Golgi remain elusive. Here, we demonstrate that Golgi-localized CDC42 effectors might be involved in FGD1-mediated post-Golgi transport, probably through coordination of Golgi membrane and cytoskeleton dynamics. Overexpression of effector-specific CDC42 mutants (exhibiting preferential affinities for PAK1, IQGAP1, N-WASP, or PAR6) only partially rescue membrane trafficking in FGD1-deficient cells, indicating that the orchestrated activities of several downstream targets of CDC42 are required to support FGD1-mediated export from the Golgi. Our findings provide new insights into understanding the molecular mechanisms behind FGD1/CDC42-dependent transport events and uncover new targets whose potential might be explored for correction of membrane trafficking in FGDY.

## Introduction

Faciogenital dysplasia (FGDY) is a rare X-linked disorder that manifests in defects of bone development such as disproportional acromelic short stature, abnormal face shape, as well as cardiac, ocular, urogenital abnormalities and mental retardation (Aarskog, 1970; Orrico et al., 2015). FGDY is caused by loss-of-function mutations in the *FGD1* gene that encodes a 961 amino acid FGD1 protein that acts as a specific GEF for the small Rho GTPase CDC42 (Pasteris et al., 1994; Zheng et al., 1996). The predicted frequency of clinical manifestation of FGDY is about 1:25,000 (Orrico et al., 2015). The FGD1 protein is expressed in regions of active osteogenesis in developing long bones (Gorski et al., 2000). In humans, the highest levels of FGD1 expression have been observed in bone tissue, kidney, liver, lung, heart and brain (Pasteris et al., 1994; Olson, 1996). Thus, the pattern of postnatal FGD1 expression strongly correlates with clinical manifestations of FGDY. Moreover, the FGD1/CDC42 signaling machinery has an important role in osteogenetic differentiation in hMSC and may persist throughout adult life (Gao et al., 2011). Similarly to other genetic diseases, there is no specific treatment for patients with FGDY, surgical intervention being the only option to correct some abnormalities and increase the quality of life.

Although FGD1 was detected at the Golgi (Estrada et al., 2001; Egorov et al., 2009), the mechanism by which the FGD1/CDC42 machinery regulates the transport of cargo at the Golgi apparatus remains unclear. We reported previously that FGD1 silencing strongly suppressed CDC42 activity at the membranes of *trans*-Golgi network (TGN). As a result, formation of post-Golgi transport intermediates was compromised, apparently due to an impairment of TGN membrane extension along microtubules (Egorov et al., 2009). In particular, this interaction is important for docking of nascent carriers to the microtubules (MTs) that trigger trafficking events. The failure of FGD1 to coordinate membrane and cytoskeletal dynamics at the Golgi could explain the membrane transport delay induced by loss-of-function *FGD1* mutations and might be considered as one of the key mechanisms of FGDY pathogenesis.

Given the importance of the FGD1/CDC42 machinery in regulating post-Golgi membrane transport, we investigated whether and to what extent the downstream targets of Golgi-localized CDC42 mediate FGD1-dependent signal transduction at the Golgi (McCallum et al., 1998; Matas et al., 2004; Cau and Hall, 2005; Valente et al., 2012; Matsui et al., 2015). Our findings indicate that suppression of either PAK1 or IQGAP1 genes resembles the inhibitory effects of FGD1 silencing on post-Golgi transport, while their activation only partially overrides the TGN transport block induced by FGD1 deficiency. Expression of effector-specific CDC42 mutants revealed that other downstream components of the FGD1/CDC42 signaling pathway including N-WASP and PAR6 might be involved in FGD1-mediated trafficking events. Further characterization of the proposed signaling pathways may help to uncover the key druggable molecules with therapeutic potential for FGDY.

## Results

**FIGURE 1:**
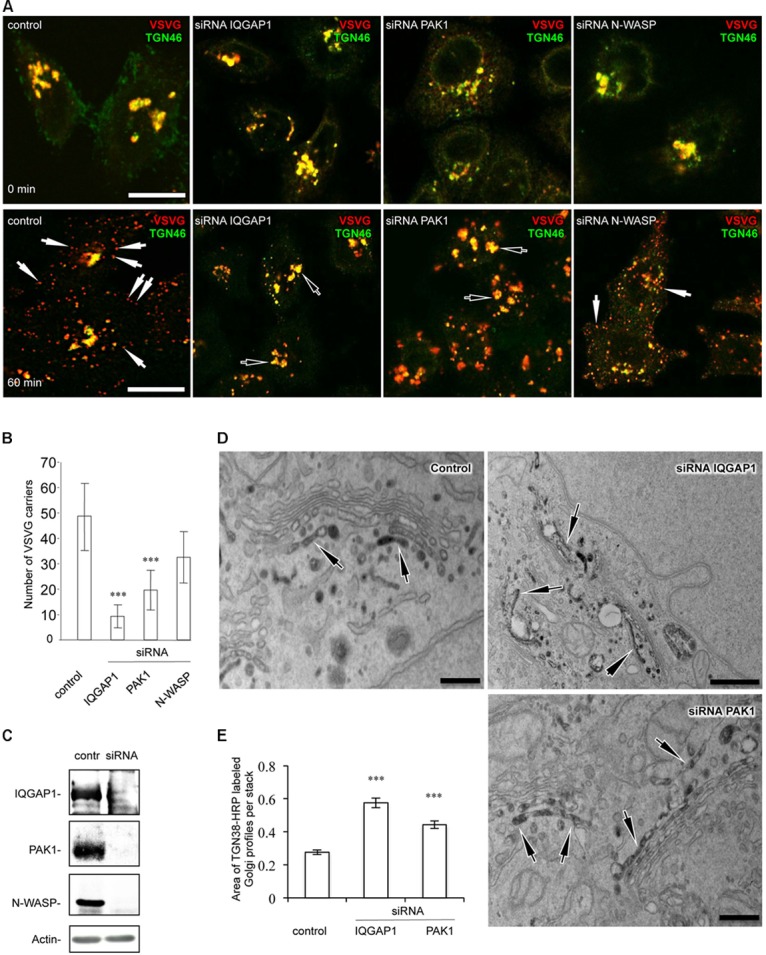
Down-stream targets of CDC42 regulate post-Golgi transport in HeLa cells. Cells were treated with either control non-targeting duplex or specific siRNAs of IQGAP1, PAK1, and N-WASP for 72 h. Cells were then processed for evaluation of post-Golgi trafficking **(A,B)**, efficiency of silencing **(C)**, or TGN morphology **(D,E)**. **(A)** After the 20°C block, VSVG accumulated at the Golgi level where it overlapped with the TGN46 marker (top row). Release of the secretory material from the Golgi (32°C 60 min; see bottom row) in the presence of 0.5% tannic acid resulted in the accumulation of VSVG carriers (arrows) in control and N-WASP-depleted cells, while most VSVG still remained in the Golgi (empty arrows) in IQGAP1- and PAK1-silenced cells. Scale bar, 15 μm. **(B)** Quantification of VSVG-containing carriers after 60 min of release from the 20°C block (*n* = 30 cells; ^∗∗∗^*p* < 0,001, *t*-test). **(C)** Western blotting showed the efficient reduction in the expression of corresponding proteins after treatment with siRNAs for N-WASP, IQGAP1, and PAK1. The level of actin was used as a loading marker. **(D)** Control, IQGAP1 or PAK1-silenced cells were transfected with TGN38-HRP, fixed and subsequently processed for electron microscopy analysis (see methods). Control HeLa cells showed the TGN-38-HRP-associated DAB precipitate in several tubular and rounded profiles at the *trans* side of the Golgi stack. Unlike control cells, the silencing of either the IQGAP1 gene or the PAK1 gene resulted in a much larger TGN, comprised of numerous additional labeled cisternae and tubular profiles. Scale bar: control and siRNA PAK1 200 nm, siRNA IQGAP1 500 nm. **(E)** Quantification of the increase in the area of the TGN compartment in IQGAP1- and PAK1-silenced cells (*n* = 20 stacks; ^∗∗∗^*p* < 0.001, *t*-test).

To determine whether Golgi-associated CDC42 targets such as IQGAP1, PAK1 and N-WASP operate in FGD1/CDC42-dependent trafficking events, we assessed the ability of these proteins to influence post-Golgi transport by monitoring constitutive export. We reasoned that CDC42 effectors whose suppression would cause the aberrant FGDY-like “secretory” phenotype (Egorov et al., 2009) are likely to be involved in FGD1/CDC42-medited regulation of trafficking events at the Golgi. To address this issue, we silenced genes of interest in HeLa cells with specific siRNAs and analyzed the impact of gene suppression on post-Golgi transport of VSVG, as previously described (Polishchuk et al., 2003). VSVG was synchronized within the TGN using a 20°C block. We found that VSVG strongly co-localized with the *trans*-Golgi marker TGN46 in both control and IQGAP1-, PAK1-, or N-WASP-depleted cells at the end of the 20°C temperature block (Figure 1A, upper row; see Figure 1C for silencing efficiency), indicating that suppression of these CDC42 effectors did not impact on the delivery of cargo proteins to the TGN. Release from the 20°C block triggered the formation of numerous VSVG-containing carriers from the Golgi apparatus in control cells. To block the fusion of carriers with the plasma membrane (which facilitates quantification), 0.5% tannic acid treatment was employed (Egorov et al., 2009). Unlike control cells, where numerous post-Golgi carriers formed within 1 h after the release of the 20°C block, IQGAP1- or PAK1-depleted cells exhibited a significant reduction in the number of TGN-derived VSVG carriers (Figure 1A, bottom row). Morphometric analysis confirmed that suppression of either IQGAP1 or PAK1 resulted in a significant decrease of VSVG-containing post-Golgi carriers (Figure 1B). In contrast, inhibition of N-WASP did not significantly affect the post-Golgi transport of VSVG (Figure 1A,B) indicating that N-WASP may operate mainly in a different transport route from the Golgi (Matas et al., 2004; Egea et al., 2013).

The inhibitory impact of IQGAP1 and PAK1 depletion on export from the TGN was confirmed by electron microscopy analysis. The general morphology of the TGN in HeLa cells lacking either PAK1 or IQGAP1 proteins were assessed using TGN38-HRP transfection (Egorov et al., 2009). The inspection of thin sections revealed a TGN38-HRP-associated DAB precipitate, which decorated the lumen of both tubular and round membrane profiles in the TGN area of control HeLa cells (Figure 1D). The silenced cells exhibited much longer and larger TGN profiles with 2–3 labeled cisternae in some Golgi stacks (Figure 1D,E). This phenotype indicates significantly reduced consumption of TGN membranes in both IQGAP1- and PAK1-deficient cells due to a delay of protein export at the level of the TGN. In summary, our observations suggest that suppression of the CDC42 downstream targets IQGAP1 and PAK1 inhibits export from the Golgi in a manner similar to what is observed in FGD1-deficient cells (Egorov et al., 2009).

**FIGURE 2:**
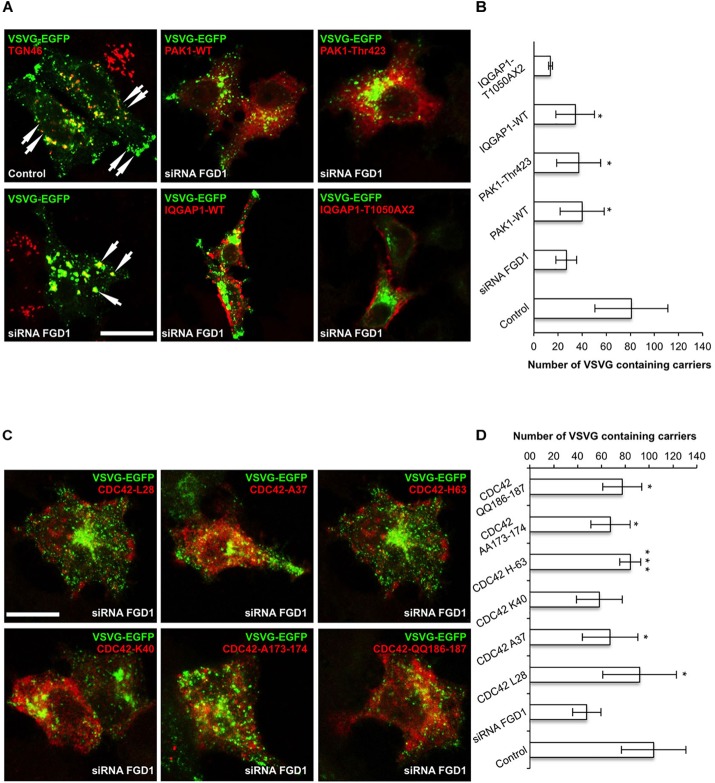
CDC42 effectors partially rescue post-Golgi transport of VSVG in FGD1-deficient HeLa cells. HeLa cells were treated with targeted FGD1 siRNA and co-transfected with VSVG-GFP and a plasmid encoding CDC42 effector (indicated at the corresponding panel). Cells were subsequently exposed to the 20°C block for 2 h and then shifted to 32°C for 60 min in the presence of tannic acid. **(A)** Expression of either the PAK1 or the IQGAP1 mutant partially rescued the VSVG-GFP transport in FGDY cells; **(B)** Quantification of post-Golgi VSVG-GFP- containing carriers per cell in the experiments shown in panel **A** (*n* = 30 cells; ^∗^*p* < 0.005, *t*-test). **(C)** Co-expression of VSVG-GFP and different constitutively active CDC42 mutants (see Table 1) partially rescued transport of VSVG-GFP in FGD1-silenced cells. **(D)** Quantification of post-Golgi VSVG-GFP-containing carriers per cell in the experiments shown in panel **C** (*n* = 30 cells; ^∗∗∗^*p* < 0.001, ^∗^*p* < 0.005, *t*-test). Scale bars, 20 μm.

To further test the impact of IQGAP1/PAK1 proteins on the FGD1-dependent mechanism of post-Golgi transport, we investigated their ability to rescue VSVG trafficking in FGD1-deficient cells. To this end, FGD1-silenced HeLa cells were co-transfected with VSVG-EGFP and plasmids encoding either PAK1-wt or PAK1-Thr423 (a constitutively active mutant) (Jaffer and Chernoff, 2002), and VSVG transport was analyzed upon a release from the 20°C block as described above. The loss of FGD1 function led to a delay of VSVG-GFP transport, while expression of PAK1-wt and PAK1-Thr423 resulted in a moderate increase in the number of post-Golgi VSVG-positive transport carriers in FGD1-deficient cells (Figure 2A,B). It appears that overexpression of PAK1 rescues post-Golgi trafficking to some extent but cannot completely overcome the transport block at the TGN induced by FGD1 knockdown.

We also evaluated the ability of IQGAP1 to facilitate protein export from the TGN in FGD1-silenced cells using the same experimental strategy. Expression of IQGAP1-WT induced a modest but significant recovery of VSVG export from the Golgi in FGD1-deficient cells (Figure 2A,B). To show that the IQGAP1-mediated rescue of trafficking in FGD1-deficient cells indeed requires CDC42, we used the IQGAP1 mutant (IQGAP1-T1050AX2), which fails to interact with its upstream activator CDC42/RAC1 (Fukata et al., 2002). We observed that expression of this particular IQGAP1 mutant was unable to rescue VSVG transport in FGD1-silenced cells even after 60 min of release from the TGN (Figure 2A,B). These findings demonstrate that IQGAP1 might potentially operate as a FGD1/CDC42 signal conductor to regulate transport from the Golgi. However, none of these downstream CDC42 effectors were sufficient alone to completely restore trafficking in FGD1-deficient cells.

To further characterize the FGD1 signaling pathway that is mediated by CDC42 effectors, we used constitutively active CDC42 mutants, which are defective in binding to different downstream targets of CDC42 (Li et al., 1999; Guo et al., 2010; See Table 1). We first tested whether expression of the CDC42/A37 mutant, which is defective in interacting with PAK1, could overcome the transport block induced by FGD1 loss-of-function using the traffic pulse protocol (Figure 2C,D). We noted a significant (but not full) recovery of VSVG-EGFP post-Golgi transport in FGD1-deficient cells expressing this CDC42 mutant. Thus, PAK1 does not appear to operate as an indispensible effector in post-Golgi trafficking events that are regulated through the FGD1/CDC42 axis. This observation is in line with a previous report showing a limited ability of FGD1 to regulate the activity of PAK1 (Nagata et al., 1998).

**Table 1:** Summary of the binding capacity of Cdc42 mutants to effectors.

CDC42 mutants	Effector binding			
	
	PAK1	N-WASP	IQGAP1	PAR6B
L28	++++	+++	++++	++
A37	-	+++	+	-
K40	+	-	+++	-
H63	+	++++	-	++
AA173-174	++++	++	+++	-
QQ186-187	+++++	+++++	+++	+


*+, binding to the indicated effectors; -, no binding activity; Li et al. (1999) and Guo et al. (2010).*

Another mutant, CDC42/H63, which is defective in binding to IQGAP1, had a similar impact. Overexpression of this mutant in FGD1-deficient cells showed a moderate improvement in VSVG-EGFP transport (Figure 2C,D), indicating that CDC42 can sustain post-Golgi trafficking through other effectors. Indeed, this mutant binds PAR6, N-WASP and to some extent PAK1 (Table 1).

Next we tested whether expression of the CDC42 mutants, which do not interact with N-WASP/PAR6 (K40) or with PAR6 (AA173-174), could restore transport in FGD1-deficient cells (Figure 2C,D). We observed no statistically significant recovery of VSVG-GFP post-Golgi transport in FGD1-silenced cells after K40 expression and only a slight increase (10.3%) in trafficking in the case of CDC42-AA173-174 expression. This suggests that FGD1/CDC42-dependent post-Golgi trafficking might require N-WASP and PAR6, whose roles in this process have to be further investigated.

Finally, we tested CDC42 constitutively active mutants that have no binding limitations toward any of the effectors (L-28 and QQ186-187). Overexpression of these mutants resulted in significant recovery of VSVG trafficking in FGD1-depleted cells. Interestingly, expression of the QQ186-187 mutant, which has a lower binding affinity for PAR6, was less efficient in recovering VSVG transport compared to CDC42/L28. This implies that only a CDC42 form that is fully active toward all downstream effectors is able to completely rescue the Golgi export block caused by FGD1 loss-of-function (Figure 2C,D). Thus, the recovery post-Golgi trafficking in FGD1-deficient cells appears to require the synergistic action of several CDC42 effectors at a certain ratio. Alternatively, the limited effect on the transport by some of the mutants might be due to their weak association with the Golgi apparatus. Additional experiments will be necessary to fully characterize the consequences of these interactions.

**FIGURE 3:**
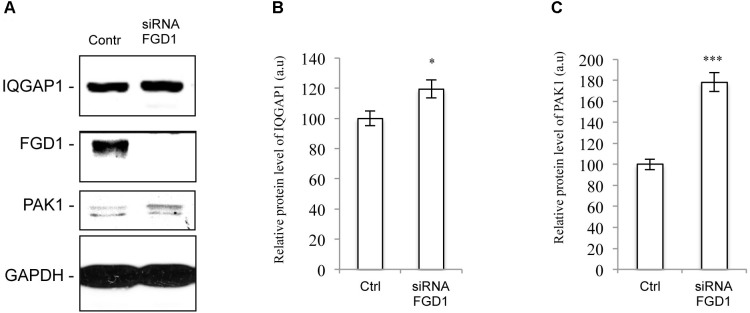
FGD1 silencing causes an increase in PAK1 and IQGAP1 expression levels. HeLa cells were silenced with FGD1-specific siRNA for 72 h, subsequently lysed, subjected to SDS–PAGE and labeled with antibodies of interest. **(A)** Immunoblots of FGD1, IQGAP1, and PAK1 in both control and FGD1-silenced cells. **(B,C)** Quantification showing the increase in IQGAP1 **(B)** and PAK1 **(C)** levels in FGD1-deficient cells (*n* = 3 experiments; ^∗∗∗^*p* < 0.001, ^∗^*p* < 0.005, *t*-test).

**FIGURE 4:**
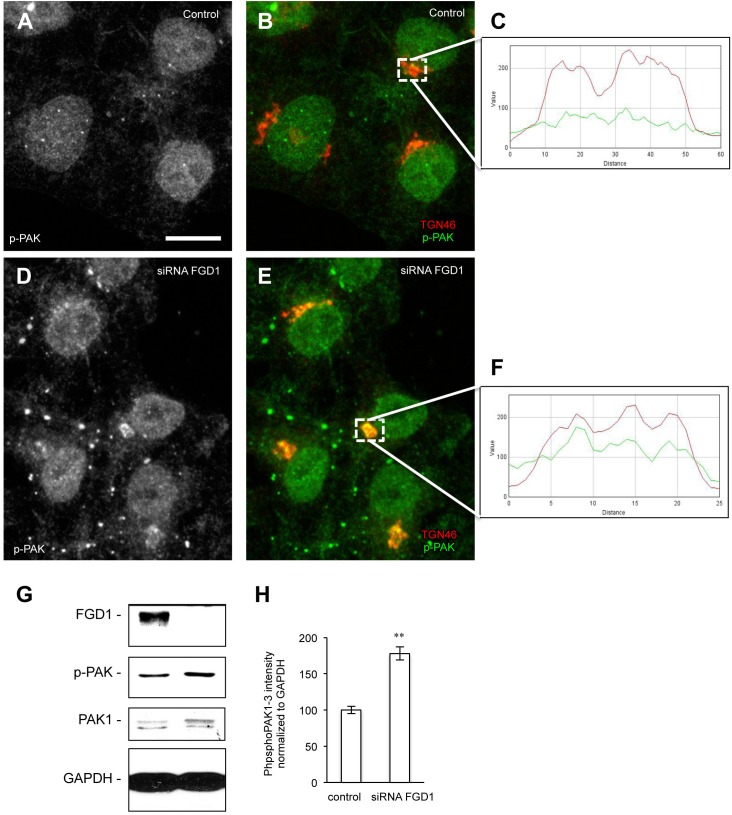
Analysis of phospho-PAK distribution in FGD1-deficient cells. **(A–F)** Hela cells were silenced with FGD1-specific siRNA for 72 h and stained with antibodies against phospho-PAK (p-PAK) and TGN46. In contrast to control cells **(A,B)**, FGD1-deficient cells exhibited substantial co-localization of p-PAK with TGN46 **(D,E)**. The quantification of PAK and TGN46 co-localization in dashed boxes is shown in control **(C)** and FGD1-deficient cells **(F)**. **(G)** Immunoblots showing FGD1, p-PAK, and PAK1 levels in control and FGD1-silenced cells. Note the increase in p-PAK in FGD1-deficient cells. **(H)** Quantification showing the increase of p-PAK in FGD1-deficient cells (*n* = 3 experiments; ^∗∗^*p* < 0.01, *t*-test). Scale bar, 20 μm.

To further explore possible mechanisms downstream of FGD1/CDC42 signaling at the Golgi, we assessed the levels of CDC42 effectors (PAK1/IQGAP1) under conditions of FGD1 silencing. Surprisingly, Western blot revealed that both PAK1 and IQGAP signals increased in FGD1-deficient cells by 78 and 19.5%, respectively (Figure 3A–C). Using an antibody that specifically recognizes the phosphorylated forms of PAK1-3, we found that Golgi-associated levels of phospho-PAK were elevated as judged by immunofluorescence inspection (Figure 4A–F). Taking into account that only PAK1 (but not PAK2/3) resides at the Golgi, we assume that the increase in phospho-PAK in the TGN46 compartment reflects activation of PAK1 at the Golgi in FGD1-deficient cells (Figure 4G,H). It appears that PAK1 becomes more active at the Golgi in the absence of functional FGD1. Thus, the up-regulation/activation of CDC42 might explain a weak compensatory response supporting cell homeostasis in conditions of a mutated FGD1 protein. This response, however, does not appear to be sufficient for recovery of normal post-Golgi trafficking rates.

**FIGURE 5:**
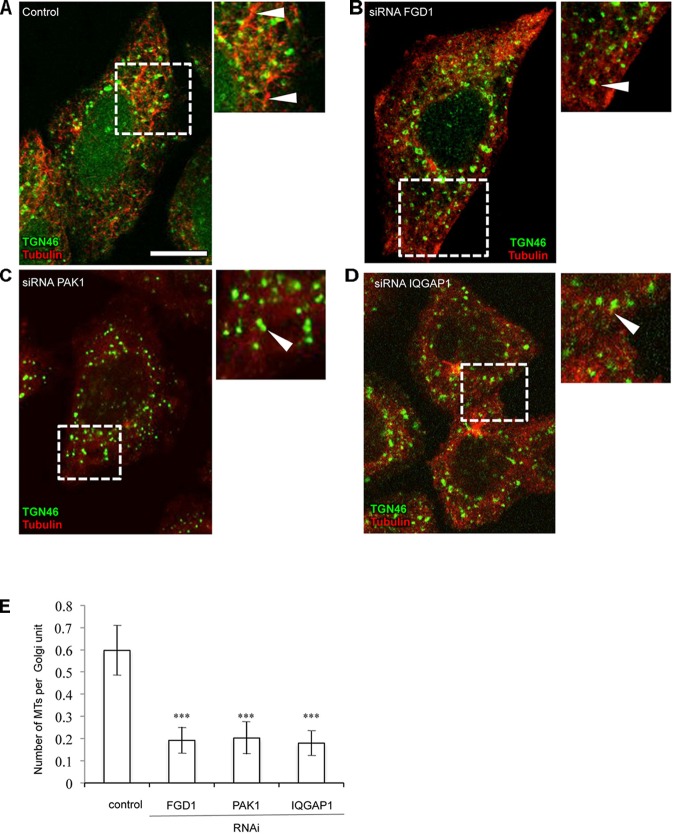
Suppression of FGD1 and CDC42 effectors impairs microtubule growth from the Golgi. HeLa cells were treated with siRNAs specific for FGD1, PAK1, or IQGAP1 for 72 h and treated with 30 μM nocodazole for 3 h to induce the MT depolymerization. Then, nocodazole was washed out for 5 min to initiate MT recovery and the cells were fixed and stained for tubulin and TGN46. **(A)** The control experiment shows the formation of the MT “asters” growing from single Golgi elements (arrowheads). **(B–D)** A delay in MT recovery was detected in case of FGD1 **(B)**, PAK1 **(C)**, and IQGAP1 **(D)** silencing. Images corresponding to the dashed box show details of MT and Golgi organization. Arrowheads indicate Golgi units with little or no associated MTs. **(E)** Quantification showing a significant reduction in the number of microtubules forming per single Golgi unit in FGD1-, PAK1-, and IQGAP-silenced cells (*n* = 30 cells; ^∗∗∗^*p* < 0.001, *t*-test). Scale bar, 15 μm.

Driven by the hypothesis that FGD1 could function in the signaling machinery that regulates the interaction between nascent TGN membranes and MTs (Egorov et al., 2009), we investigated the involvement of FGD1 and CDC42 effectors in both the nucleation and growth of the Golgi-associated subset of MTs in HeLa cells. To this end, we employed a well-known approach based on the regrowth of MTs in interphase cells after their nocodazole-induced depolymerization (Efimov et al., 2007). Immunofluorescence revealed that control cells quickly recovered the Golgi-associated pool of MTs 2–10 min after nocodazole washout (Figure 5A,E). By contrast, there was a significant delay of MT recovery in the case of FGD1 suppression since only a few MT profiles emerged from the Golgi elements in FGD1-silenced cells (Figure 5B,E). This effect of FGD1 depletion on Golgi-associated MT growth might contribute to inhibiting the formation of post-Golgi carriers, which exit from the TGN along MT tracks (Polishchuk et al., 2003; Egorov et al., 2009). Similarly to FGD1 knockdown, the silencing of either PAK1 or IQGAP1 by specific siRNAs affected the efficiency of MT regrowth from the Golgi units (Figure 5C–E) suggesting that both CDC42 effectors regulate the dynamics of MTs emerging from the Golgi. Importantly, in these experiments we found that the rate of MT growth from the MTOC was not affected, implying the involvement of FGD1, PAK1 and IQGAP in MT dynamics operates only at Golgi stacks.

**FIGURE 6:**
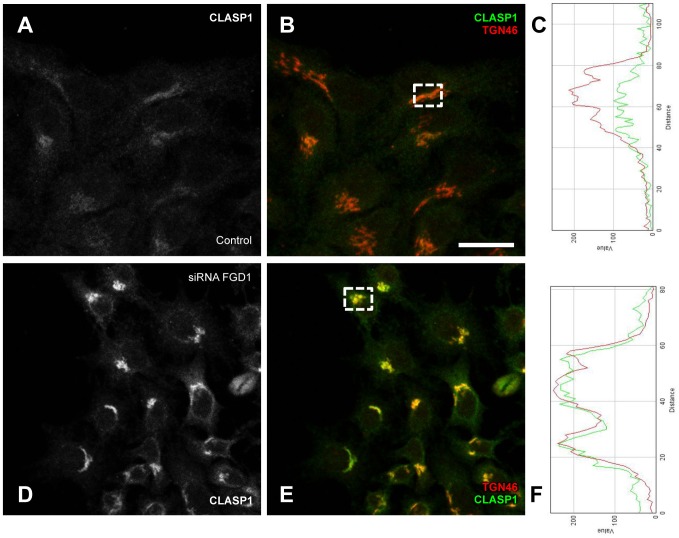
Analysis of CLASP1 distribution in FGD1-deficient cells. HeLa cells were transfected with FGD1-specific siRNAs and stained with antibodies against CLASP1 and TGN46. Control cells **(A,B)** exhibited a faint CLASP1 signal at the Golgi, while CLASP1 levels significantly increased in FGD1-deficient cells **(D,E)**. Quantification **(C,F)** showing CLASP1 and TGN46 fluorescence in the areas outlined by dashed boxes in panels **B** (control cells) and **D** (FGD1-deficient cells). Scale bar, 28 μm.

The function, stabilization and dynamics of MTs are tightly regulated by numerous MT-associated proteins. One of these is the well-characterized MT non-motor plus-end-binding protein CLASP (cytoplasmic linker associated protein (1) that has been shown to drive nucleation of non-centrosomal MTs originating from the TGN (Efimov et al., 2007). We reasoned that the dynamics of CLASP1 at the Golgi might be affected by FGD1 loss-of-function. Confocal microscopy showed a very clear but faint CLASP1 signal at the Golgi membranes in control cells (Figure 6A–C). By contrast, FGD1-silenced cells exhibited a strong increase in CLASP1 levels at the Golgi (Figure 6D–F) indicating that molecular coordination of Golgi membranes and MT dynamics are likely to be impaired by the loss of FGD1.

## Discussion

Our findings indicate that the impact of FGD1 on post-Golgi trafficking is indeed mediated by CDC42 effectors. However, none of the CDC42 effectors appears to be absolutely indispensible: rather, it is the orchestrated activity of several such effectors that is required to support export of cargo proteins from the TGN. We have shown that direct Golgi-associated downstream targets of CDC42, such IQGAP1 and PAK1, are involved in regulating constitutive post-Golgi membrane transport. PAK1 has been already reported to operate in the fission of post-Golgi carriers from the TGN (Valente et al., 2012), while the involvement of IQGAP1 in post-Golgi trafficking has not yet been observed despite a well-documented association of IQGAP with the Golgi (McCallum et al., 1998). Our observations suggest that FGD1, IQGAP1 and PAK1 are required (i) to support formation of post-Golgi carriers and (ii) to allow nucleation of Golgi-associated MTs, which are used by post-Golgi carriers as highways to reach their target destinations (Polishchuk et al., 2003; Egorov et al., 2009). However, both IQGAP and PAK1 exhibited a limited ability to restore the impaired TGN export in FGD1-deficient cells when expressed alone. Indeed, the impact of their overexpression on post-Golgi trafficking in the absence of FGD1 appears to be incomplete, probably due to the lack of activity of other effectors coordinated by the FGD1/CDC42 machinery or by their weaker association with TGN membranes when CDC42 is missing. On the other hand, we found that the levels of both IQGAP1 and PAK1 were increased in cells lacking FGD1.

This indicates that cells might try to compensate for the loss of FGD1 through the up-regulation of the CDC42 effectors PAK1 and IQGAP1. These compensatory mechanisms may target PAK1 and IQGAP1 through FGD1 independent signaling pathways in an attempt to circumvent the FGD1 deficiency. In this regard, FGD1 has been shown to play a limited role in PAK1 activation (Nagata et al., 1998) indicating that additional mechanisms might be involved to stimulate PAK1 activity at the Golgi. However, such activation does not appear to be sufficient to recover post-Golgi transport, probably because other components of the FGD1/CDC42-controlled molecular network remain inactive or cannot be recruited to the Golgi.

The complexity of FGD1-regulated post-Golgi transport might imply the coexistence of several CDC42-dependent signaling pathways, some of which may include N-WASP as an effector. This hypothesis is supported by our findings showing that CDC42 mutants with impaired N-WASP binding have a limited capacity to support VSVG export from the Golgi in FGD1-deficient cells. N-WASP regulates actin dynamics and the formation of transport carriers at the Golgi (Egea et al., 2013). We cannot rule out that actin-based mechanisms of post-Golgi carrier formation are also controlled by the FGD1/CDC42 machinery. WASP, as a CDC42 target, might be a part of this mechanism as well, but our RNAi results suggest that WASP is probably indispensable for post-Golgi trafficking.

**FIGURE 7:**
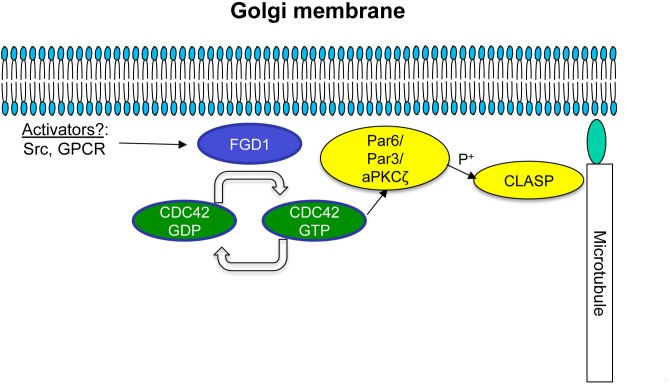
Schematic representation of possible FGD1/CDC42-dependent coordination of microtubule dynamics at the Golgi membranes. A Golgi pool of FGD1 promotes GTP binding and, hence, activation of CDC42. The GTP-bound form of CDC42 activates the PAR6/PAR3 complex that in turn mediates aPKC-dependent phosphorylation of CLASPs, facilitating nucleation and stabilization of microtubules growing from the TGN. In conditions of FGD1 deficiency, signal transduction from CDC42 to CLASP might be affected leading to an increase in CLASP levels at the Golgi resulting in a delay in MT dynamics. We speculate that impaired post-Golgi transport of secretory proteins in FGDY might be explained in part by this mechanism, including a lack of coordination between growing MTs and nascent Golgi transport intermediates.

Our findings also suggest that one of the plausible mechanisms by which FGD1 regulates post-Golgi transport requires the stabilization of a Golgi-emanating subset of MTs, which serve as tracks for the formation of post-Golgi carriers and for their further translocation to the target membrane (Polishchuk et al., 2003; Egorov et al., 2009). Indeed, nucleation of MTs at the Golgi membranes was strongly inhibited by silencing of FGD1 or by suppression of the CDC42 effectors IQGAP and PAK1. MT assembly at Golgi membranes is driven by MT-associated proteins such as CLASPs (Efimov et al., 2007; Miller et al., 2009). It has been shown recently that the dynamics of CLASP2 association with the Golgi is regulated by a novel protein complex, PAR6/PAR3/aPKC, that has CDC42 and PAR6 as targets (Matsui et al., 2015). This protein complex controls CLASP2 phosphorylation at sites responsible for its interaction with GCC185 at the TGN (Matsui et al., 2015). Disruption of this PAR6 complex results in aberrant CLASP accumulation at the Golgi (Matsui et al., 2015). Notably, FGD1 knockdown causes a similar increase in CLASP association with Golgi membranes (see Figure 6). Thus, it is tempting to speculate that the missing link between the FGD1/CDC42 machinery and MTs could be constituted by the PAR/PKC-CLASP complex (Figure 7). In this scenario, the lack of FGD1 activity might impact on the PAR6 complex and, hence, further affect the dynamics of CLASP. This, in turn, would cause either MT instability or a low rate of MT regrowth, thus affecting the formation and translocation of Golgi-derived transport carriers and post-Golgi trafficking in general. Indeed, abnormal CLASP accumulation and impaired nucleation of MTs at the Golgi in FGD1-deficient cells supports this hypothesis. We also found that loss of interaction with PAR6 reduces the ability of constitutively active CDC42 mutants to rescue transport in FGD1-deficient cells. This suggests that PAR6 is involved in the FGD1/CDC42 pathway that regulates MT dynamics at TGN membranes.

Further studies of the molecular players involved in FGD1/CDC42-dependent regulation of post-Golgi trafficking represents a challenging task in finding a cure for FGDY. Although we found that activation of some CDC42 effectors may partially compensate for FGD1 deficiency, none of these effectors alone circumvent the consequences of FGD1 loss. Therefore, drugs or treatments that stimulate multiple FGD1/CDC42 targets have to be considered for eventual therapeutic approaches. Furthermore, considering the abundance of FGD1 in bones and its importance for the transport of bone proteins (Egorov et al., 2009), pharmacological activation of FGD1/CDC42 effectors deserves serious evaluation as a potential strategy to facilitate bone regeneration after damage or surgery. In this context, significant efforts will be required to identify key druggable nodes in the FGD1/CDC42 signaling pathway(s) operating in the bone-specific post-Golgi secretory pathway(s).

## Materials and Methods

### Antibodies and Reagents

The following antibodies were used: anti-TGN46 1:1000 (IF) (AbD Serotec, United Kingdom); anti-Giantin 1:500 (IF) (Abcam, United Kingdom); anti-myc 1:250 (IF); anti-flag 1:250 (IF); anti-HA 1:500 (IF) and P5D4 Cy3-conjugated anti-VSVG 1:500 (IF) from Sigma-Aldrich (Italy); anti-N-WASP 1:1000(WB); anti-IQGAP1 1:1000(WB); phosphoPAK1-3 1:500 (IF), 1:1000 (WB); and PAK1 (N-20) 1:500 (IF), 1:1000 (WB) were purchased from Santa Cruz Biotechnology (United States); anti-FGD1 1:1000 (WB) made by G. Di Tullio (CMNS, Italy); GAPDH 1:1000 (WB), CLASP1 1:300 (IF) provided by I. Kaverina (Vanderbilt University, United States).

The following plasmids were used: IQGAP1-T1050AX2-flag (provided by K. Kaibuchi, Japan); IQGAP1-WT-myc (Columbia University); PAK1-WT-myc, PAK1-Thr423-myc (provided by M. Gimona); CDC42 mutants were tagged with HA (a gift form Y. Zheng, United States); VSVG-EGFP (provided by J. Lippincott-Schwarz, NIH/NICHD, United States).

### Cell Culture, Transfections, and Infection With VSV

HeLa cells (ATCC, United States) were cultured in DMEM (Invitrogen, Italy) supplemented with 10% FBS. Both non-targeted (control) and target-specific siRNAs were obtained from Sigma Aldrich (Italy) and transfected by Oligofectamine (Invitrogen, Italy). The efficiency of silencing was assessed for each experiment by Western blotting. Plasmid transfection was performed using Lipofectamine 2000 (Invitrogen, Italy). The infection of HeLa with VSV was performed as previously described (Polishchuk et al., 2003). The nododazole-washout assay was performed according to a published protocol (Efimov et al., 2007).

### Western Blotting

RNAi-treated HeLa cells were lysed in 50 mM Tris–HCl, pH 8.8, containing 0.2% SDS and protease inhibitor cocktail (Roche, France). Sixty micrograms of cell lysates were run on 7% SDS–PAGE and subsequently transferred to nitrocellulose. Blots were probed with specific antibodies of interest using the ECL-based detection method (Amersham Pharmacia Biotech, Piscataway, NJ, United States).

### Immunofluorescence and Confocal Microscopy

For immunofluorescence analysis, HeLa cells were fixed with 4% paraformaldehyde in phosphate-buffered saline at room temperature for 30 min, permeabilized with 0.2% saponin for 30 min, and then blocked with 2% bovine serum albumin (BSA) for 30 min. The cells were labeled with the primary antibodies and secondary antibodies of interest conjugated to Alexa Fluor 488 and 568. Cells were mounted with mowiol and analyzed on LSM 510 META or LSM710 confocal microscopes (Carl Zeiss, Germany) with the 63 × Apo NA 1.4 objective. Numbers of VSVG carriers and numbers of MTs emerging from the Golgi were quantified in confocal images using ImageJ software (NIH, United States) as described (Egorov et al., 2009). Colocalization of p-PAK or CLASP1 with TGN46 was evaluated using either ImageJ (NIH, United States) or Zen Black (Zeiss, Germany) software.

### Electron Microscopy

For electron microscopy analysis, cells of interest expressing TGN38-HRP were directly fixed with 2% glutaraldehyde (pH 7.4) and incubated with buffered 3,3′-diaminodenzidine DAB, according to Egorov et al. (2009). After dehydration the cells were embedded in Epon812. Embedded cells were sectioned with a 45° degree diamond knife (Diatome, Switzerland) using a Leica ultramicrotome (Leica, Austria). The thin 70 nm sections were imaged with a Tecnai-12 electron microscope (FEI, The Netherlands) equipped with an Ultra View CCD digital camera (Soft Imaging System, Munich, Germany).

### Statistical Analysis

All statistical data are presented as a mean value ± standard deviation. The unpaired Student *t-test* was used to calculate differences between two sets of data. Statistical significance between different sets of data are indicated in the text and figures as follows: ^∗^*p* < 0.05; ^∗∗^*p* < 0.01; and ^∗∗∗^*p* < 0.001. All results are representative of three independent experiments.

## Author Contributions

All authors listed have made a substantial, direct and intellectual contribution to the work, and approved it for publication.

## Conflict of Interest Statement

The authors declare that the research was conducted in the absence of any commercial or financial relationships that could be construed as a potential conflict of interest.
